# Multimodal Imaging, OCT B-Scan Localization, and *En Face* OCT Detection of Macular Hyperpigmentation in Eyes with Intermediate Age-Related Macular Degeneration

**DOI:** 10.1016/j.xops.2022.100116

**Published:** 2022-01-24

**Authors:** Rita Laiginhas, Jeremy Liu, Mengxi Shen, Yingying Shi, Omer Trivizki, Nadia K. Waheed, Giovanni Gregori, Philip J. Rosenfeld

**Affiliations:** 1 Department of Ophthalmology, Bascom Palmer Eye Institute, University of Miami Miller School of Medicine, Miami, Florida; 2 New England Eye Center, Tufts Medical Center, Boston, Massachusetts

**Keywords:** Age-related macular degeneration, Color fundus imaging, *En face*, Hyperpigmentation, Hyperreflective foci, Hypotransmission, AMD, age-related macular degeneration, CFI, color fundus image, FAF, fundus autofluorescence, FOV, field of view, hyperTD, hypertransmission defect, hypoTD, hypotransmission defect, iAMD, intermediate age-related macular degeneration, NIR, near infrared, RPE, retinal pigmented epithelium, SS, swept-source

## Abstract

**Purpose:**

Multimodal imaging was used to identify and characterize the cause of hyperpigmentation seen on color fundus images (CFIs) of eyes with intermediate age-related macular degeneration (iAMD).

**Design:**

Retrospective review of a prospective study.

**Participants:**

Patients with iAMD.

**Methods:**

Color fundus images with macular hyperpigmentation were compared with same-day images obtained using fundus autofluorescence (FAF), near infrared reflectance (NIR), and swept-source (SS) OCT imaging. Two SS OCT *en face* slabs were generated: a retinal slab to identify hyperreflective foci within the retina and a slab from beneath the retinal pigment epithelium (RPE; the sub-RPE slab) that was used to detect regions that cause decreased light transmission into the choroid, also known as hypotransmission defects. All images were registered to allow for qualitative comparisons by 2 independent graders.

**Main Outcome Measures:**

Comparison between foci of macular hyperpigmentation seen on CFIs with the detection of these regions on FAF, NIR, and SS OCT *en face* images.

**Results:**

Compared with CFIs, FAF imaging seemed to be the least sensitive method for the detection of hyperpigmentation, whereas NIR and SS OCT imaging reliably detected these hyperpigmented areas. Although NIR imaging detected most of the hyperpigmentation seen in CFIs, SS OCT imaging detected all the areas of hyperpigmentation and anatomically localized these areas by using both *en face* and B-scan images. *En face* OCT slabs of the retina and sub-RPE region were registered to the CFIs, and areas of hyperpigmentation were shown to correspond to hyperreflective foci in the retina and regions of thickened RPE seen on OCT B-scans. Although both hyperpigmentation and early atrophic lesions appeared bright on NIR imaging, *en face* SS OCT imaging was able to distinguish these lesions because hyperpigmentary changes appeared dark and early atrophic lesions appeared bright on the sub-RPE slab.

**Conclusions:**

*En face* OCT imaging in conjunction with OCT B-scans were able to identify and localize the hyperpigmentation seen in CFIs reliably. This hyperpigmentation was not only associated with intraretinal hyperreflective foci, but also corresponded to areas with a thickened RPE.

Hyperpigmentation in the macula of eyes with age-related macular degeneration (AMD) has been recognized as a risk factor for disease progression.^1^, ^2^, ^3^, ^4^, ^5^ In the 9-step severity scale from the Age-Related Eye Disease Study,^1^ the presence of these pigmentary deposits was recognized as one of several important risk factors for disease progression. In the simplified Age-Related Eye Disease Study severity scale,^2^ only the presence of drusen and pigmentary changes was needed to assess the overall risk of disease progression. In a subsequent clinical classification article in which AMD was divided into early, intermediate, and late stages, the presence of medium drusen alone was sufficient to qualify an eye as having early AMD, whereas the addition of pigmentary abnormalities resulted in more advanced classification of intermediate AMD (iAMD).^5^

In AMD, these areas of macular hyperpigmentation have been described as dark colored, often spiculated lesions within the macula that appear darker than the surrounding fundus on examination and on color fundus images (CFIs).^6^ Although CFIs were considered the historical gold standard for assessing risk-stratification scales in AMD, more recent imaging technologies such as autofluorescence, infrared, and OCT imaging have several advantages, such as enhanced contrast detection and the decreased influence of media opacities such cataracts. OCT imaging has the added advantage of providing depth-resolved information with the ability to localize specific anatomic changes and to associate these alterations with the stage and progression of AMD.^7^ Because of the ability of OCT imaging to provide cross-sectional imaging of the macular anatomic features in AMD, the Classification of Atrophy Meetings consensus group proposed that OCT imaging should serve as the reference standard for the diagnosis and monitoring of disease progression in AMD.^8^

In AMD, macular hyperpigmentation has been most frequently associated with the presence of intraretinal hyperreflective foci identified using OCT B-scans in eyes with nonexudative AMD.^9^, ^10^, ^11^, ^12^, ^13^ It was speculated that these hyper-reflective foci represented the aggregation of retinal pigment epithelium (RPE) cells that had migrated to the retina, a process supported by histopathologic reports of eyes with AMD.^14^ However, the relationship between hyperreflective foci and pigmentary abnormalities seen on fundus examination and CFIs is not straightforward because not all hyperreflective foci in the retina corresponded to regions of pigmentary abnormalities seen on CFIs, and not all eyes with pigmentary changes on CFIs showed hyperreflective lesions on OCT B-scans.^9^ However, in a recent study by Hammer et al,^15^ the authors used fluorescence lifetime imaging ophthalmoscopy to identify changes in the emission spectra of the RPE in eyes with AMD, and they associated hyperpigmentation seen on CFIs with hyperpigmentation associated with intraretinal hyperreflective foci and a thickened RPE.

In addition to using OCT B-scans to study eyes with AMD, we have routinely used *en face* OCT imaging to diagnose the stage and progression of AMD.^16^, ^17^, ^18^, ^19^ The advantages of *en face* OCT imaging include the ability to evaluate AMD over 2 lateral dimensions, rather than a single horizontal dimension corresponding to an OCT B-scan. Moreover, an *en face* OCT image is more easily related to the view of AMD seen with fundus examinations, CFIs, autofluorescence images, and infrared reflectance images. In addition, it is easier to review an *en face* OCT image at a glance, rather than laboriously scrolling through individual B-scans from the entire scan volume. *En face* imaging also allows for the visualization of specific anatomic layers by selecting boundary-specific slabs that encompass the anatomic layers of interest. For example, an *en face* image of the retina would include a slab with boundaries from the internal limiting membrane to the inner boundary of the RPE, whereas a subretinal slab would include a slab with the inner boundary from the RPE to a position posterior to the RPE, such as within the choroid.

By using *en face* imaging in eyes with iAMD, we demonstrated that hypertransmission defects (hyperTDs), which corresponded to areas of evolving or established macular atrophy, can be detected as bright regions within an *en face* OCT image that was derived from a sub-RPE slab with segmentation boundaries positioned 64 and 400 μm below Bruch’s membrane.^16^, ^17^, ^18^, ^19^ The RPE normally serves as a highly reflective layer that limits the penetration of light into the choroid, and hyperTDs are the result of increased penetration of light into the choroid through regions where RPE is either attenuated or absent. Of note, when we used the sub-RPE slab to detect bright foci referred to as hyperTDs, we also detected dark foci or hypotransmission defects (hypoTDs). These hypoTDs corresponded to focal regions of decreased light penetration into the choroid. Reviewing the B-scans corresponding to the location of hypoTDs on *en face* images in eyes with iAMD, we found that the hypoTDs corresponded to intraretinal hyperreflective foci and to changes along the RPE that seemed to be associated with a thickened RPE layer, and this thickened RPE appeared to block light transmission into the choroid. These hypoTDs were also associated with focal pigmentary abnormalities seen on CFIs and fundus examinations.

In this study, we used multimodal imaging of iAMD eyes with macular hyperpigmentation to demonstrate that pigmentary changes on CFIs correlate with foci of hyperreflectivity both within the retina and along the RPE.

## Methods

Participants in this retrospective case series had been prospectively enrolled into an observational swept-source (SS) OCT imaging study that included patients with iAMD^5^ at the Bascom Palmer Eye Institute. The institutional review board of the University of Miami Miller School of Medicine approved the study (protocol no., 20170512), and all patients signed an informed consent form for this prospective SS OCT natural history study. The study was performed in accordance with the tenets of the Declaration of Helsinki and complied with the Health Insurance Portability and Accountability Act of 1996.

### Imaging Protocols

Representative eyes with iAMD and macular hyperpigmentation on CFIs were included in this retrospective cases series. Each patient in the study underwent color fundus imaging using a 50° field of view (FOV) centered on the fovea (TRC-50DX; Topcon Medical Systems). Hyperpigmentation on CFIs was identified as dark-colored, often spiculated lesions within the macula that appeared darker than the surrounding fundus. At the same visit when CFI was performed, patients underwent fundus autofluorescence (FAF) imaging (30° FOV centered on the fovea; ART 25, HRA-II [Heidelberg Engineering]), near infrared (NIR) imaging (30° FOV centered on the fovea; ART 25, HRA-II), and SS OCT angiography imaging (PLEX Elite 9000; Carl Zeiss Meditec, Dublin, CA). Swept-source OCT angiography imaging included a 6 × 6-mm scan (20° FOV) centered on the fovea. This scan pattern consisted of 500 A-scans per B-scan, with each B-scan repeated twice at each position to generate both the structural and angiographic image; 500 B-scan positions were along the slow axis, resulting in a uniform spacing of 12 μm between A-scans. Each A-scan had a depth of 3 mm consisting of 1536 pixels per A-scan. The scans were reviewed for quality and signal strength, and scans with a signal strength of < 7 based on the instrument’s output or significant motion artifacts were excluded. If more than 1 scan of a given type was available at a visit, the scan with the best quality was chosen.

### Swept-Source OCT Image Processing

The SS OCT 6 × 6-mm volume scan was used to prepare 2 *en face* images: a sub-RPE slab extending 64 to 400 μm below Bruch’s membrane and a retinal slab extending from the internal limiting membrane to the inner boundary of the RPE. The *en face* sub-RPE slab was used to detect regions with increased choroidal light transmission, known as choroidal hyperTDs, and decreased choroidal light transmission, known as choroidal hypoTDs. The multilayer segmentation algorithm provided by the device was used to automatically perform the segmentation of the retina layers. This segmentation was revised and edited manually if needed. Two separate graders (R.L. and J.L.) independently evaluated all *en face* images for the presence of these transmission defects. Consensus gradings of the transmission defects were reached between the 2 graders for each lesion in each eye. Any remaining disagreement was adjudicated by a senior grader (P.J.R.). Whenever a hyperTD or hypoTD was identified on the *en face* sub-RPE slab, the corresponding B-scans were inspected to identify any structural changes that could account for these abnormalities in choroidal light transmission.

The retinal *en face* slab, which extended from the internal limiting membrane to the RPE, was used to identify hyperreflective foci within the retina using a strategy similar to the one described by Nassisi et al.^20^ These hyperreflective foci could be seen as focal bright spots on the *en face* image of the retina. Whenever a bright spot was identified on the *en face* retinal slab, the corresponding B-scans were inspected to identify any structural changes that could account for these foci of hyperreflectivity within the retina.

All images were exported as JPEG files for further analyses. The CFIs, FAF images, and NIR images were manually registered and resized using Adobe Photoshop (Adobe, Inc) to allow for qualitative comparisons of these images. The retinal vessels were used as landmarks for the manual registration.

A qualitative descriptive comparison was then performed between the hyperpigmentation seen on CFIs with FAF, NIR, and SS OCT *en face* images. The corresponding SS OCT B-scans for the regions of interest were also exported and were correlated with the multimodal fundus images.

## Results

**Figure 1:**
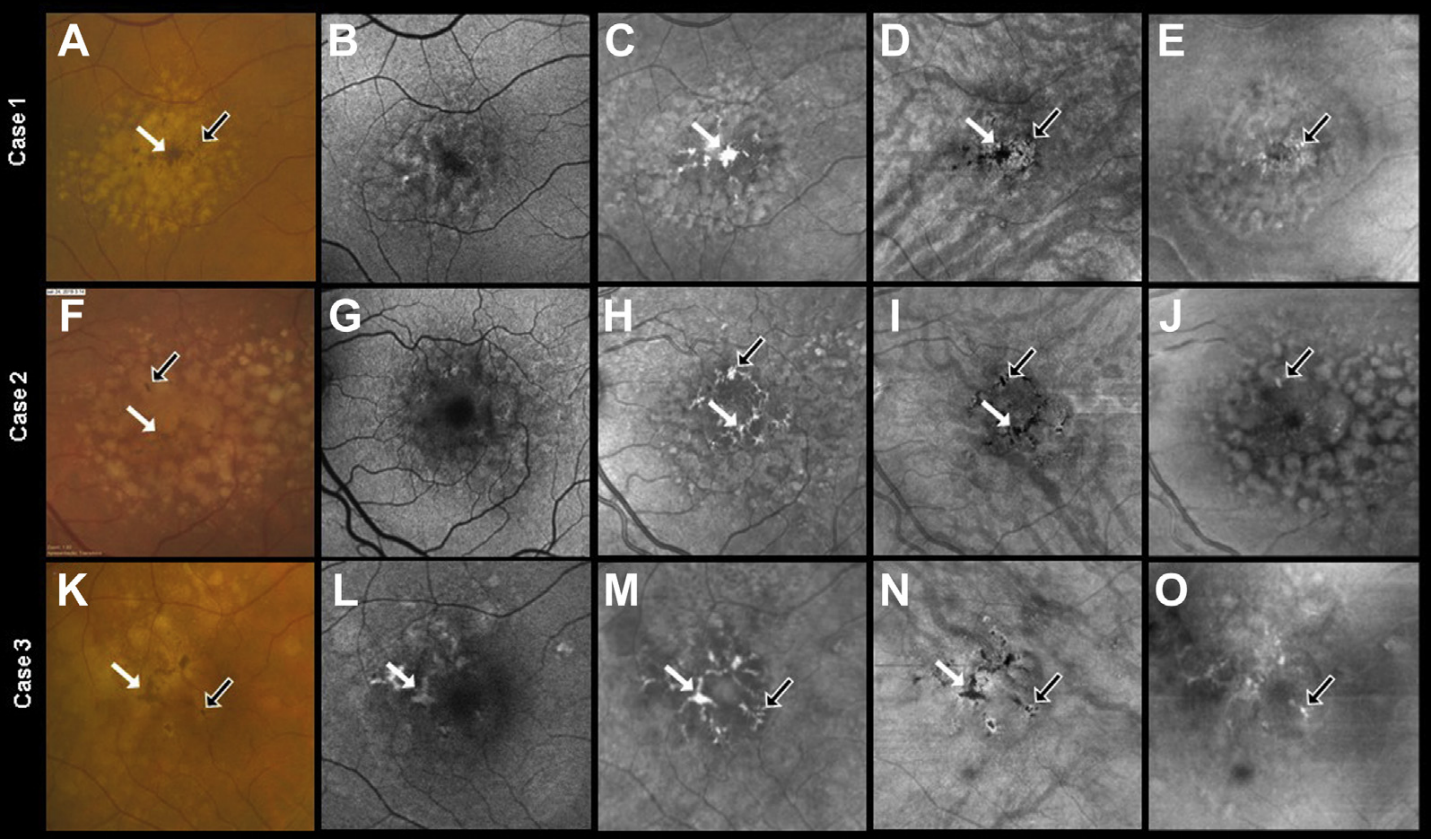
Multimodal *en face* imaging of pigmentary changes in 3 eyes from 3 patients with intermediate age-related macular degeneration: (**A**, **F**, **K**) color fundus images (CFIs), (**B**, **G**, **L**) fundus autofluorescence (FAF) images, (**C**, **H**, **M**) near infrared (NIR) images, (**D**, **I**, **N**) swept-source (SS) OCT *en face* sub–retinal pigmented epithelium (RPE) slabs, and (**E**, **J**, **O**) SS OCT *en face* retinal slabs. Foci of hyperpigmentation are seen as dark brown spots in CFIs. The white and black arrows highlight 2 of these hyperpigmentation foci. The FAF images (**B**, **G**, **L**) identified a subset of hyperpigmentary changes seen on the CFIs, whereas the NIR images (**C**, **H**, **M**) identified most of the hyperpigmentary foci seen on CFIs. The *en face* sub-RPE OCT slab (**D**, **I**, **N**) was able to show all the hyperpigmentation foci seen on CFIs. These lesions appear as dark spots on the sub-RPE slab, which correspond to hypotransmission defects (**D**, **I** , **N**, white and black arrows). In the *en face* retinal slab (**E**, **J**, **O**), bright foci (black arrows) correspond to hyperreflective foci within the retina. Of note, the burden of hyperreflective foci seen on the *en face* retinal slab (black arrows) reflect a small proportion of the hyperpigmentation foci seen on CFIs. This happens because the remaining regions of macular hyperpigmentation correspond to regions of thickened RPE that we explore further in Figures 3, 4, and 5.

**Figure 2:**
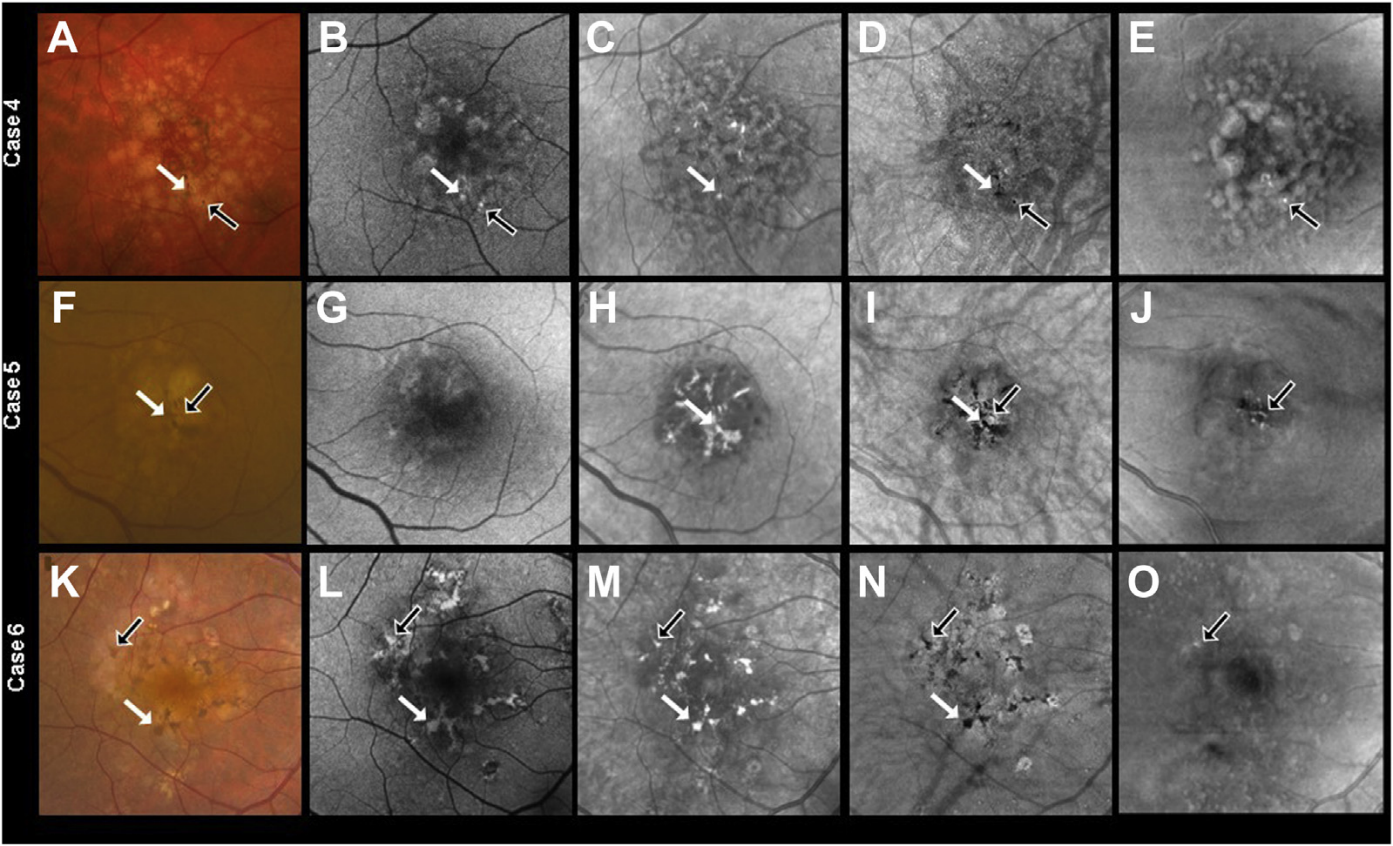
Multimodal *en face* imaging of pigmentary changes in 3 eyes from 3 patients with intermediate age-related macular degeneration: (**A**, **F**, **K**) color fundus images (CFIs), (**B**, **G**, **L**) fundus autofluorescence (FAF) images, (**C**, **H**, **M**) near infrared (NIR) images, (**D**, **I**, **N**) swept-source (SS) OCT *en face* sub–retinal pigmented epithelium (RPE) slabs, and (**E**, **J**, **O**) SS OCT *en face* retinal slabs. Foci of hyperpigmentation are seen as dark brown spots in CFIs. The white and black arrows highlight 2 of these hyperpigmentation foci. The FAF images (**B**, **G**, **L**) identified a subset of hyperpigmentary changes seen on the CFIs in patients 4 and 6 (**B**, **L**), but missed them in patient 5 (**G**). The NIR images (**C**, **H**, **M**) identified most of the hyperpigmentary foci seen on CFIs, although some clumps were missed in patients 4 and 5 (**C**, **H**). The *en face* sub-RPE OCT slab (**D**, **I**, **N**) was able to show all the hyperpigmentation foci seen on CFIs. These lesions appear as dark spots on the sub-RPE slab, which correspond to hypotransmission defects (white and black arrows). In the *en face* retinal slab (**E**, **J**, **O**), bright foci (black arrows) correspond to hyperreflective foci within the retina. Of note, the burden of hyperreflective foci seen on the *en face* retinal slab (black arrows) reflect a small proportion of the hyperpigmentation foci seen on CFIs. This happens because the remaining regions of macular hyperpigmentation correspond to regions of thickened RPE that we explore further in Figures 6, 7, and 8.

Six eyes from 6 patients (age range, 54–77 years) with iAMD and hyperpigmentation on CFIs were selected to demonstrate the localization of these pigmentary changes on OCT images. In this series, none of the images required manual editing of the segmentation boundaries. Figures 1 and 2 show the *en face* multimodal images from these patients, with the CFIs serving as the reference for hyperpigmentation. When compared with CFIs, FAF imaging seemed to be the method least likely to show the hyperpigmentation, whereas NIR and SS OCT imaging seemed to reliably detect these areas of hyperpigmentation. In NIR imaging (Figs 1C, H, M and 2C, H, M), the larger areas of hyperpigmentation appeared as bright regions; however, the smaller pigmentary foci were not always apparent when compared with CFIs. In Figures 1 and 2 (cases 1, 4, and 5), pigmentary changes seen on CFIs (see arrows) were not apparent on NIR imaging (Figs 1C and 2C, H).

Swept-source OCT *en face* imaging was able to clearly demonstrate the hyperpigmentation seen on CFI (Figs 1D, E, I, J, N, O and 2D, E, I, J, N, O). Although the sub-RPE slab (Figs 1D, I, N and 2D, I, N) identified the foci of hyperpigmentation seen on CFIs, the retinal slab was able to localize those foci of hyperpigmentation that resided within the retina (Figs 1E, J, O and 2E, J, O). Another advantage of the sub-RPE slab image is that, in contrast to the other imaging methods, foci of evolving RPE atrophy could be identified as bright spots because of the presence of hyperTDs in these areas, whereas these evolving areas of atrophy cannot be easily distinguished using any of the other imaging methods (Figs 2N and 8A, E, F).

**Figure 3:**
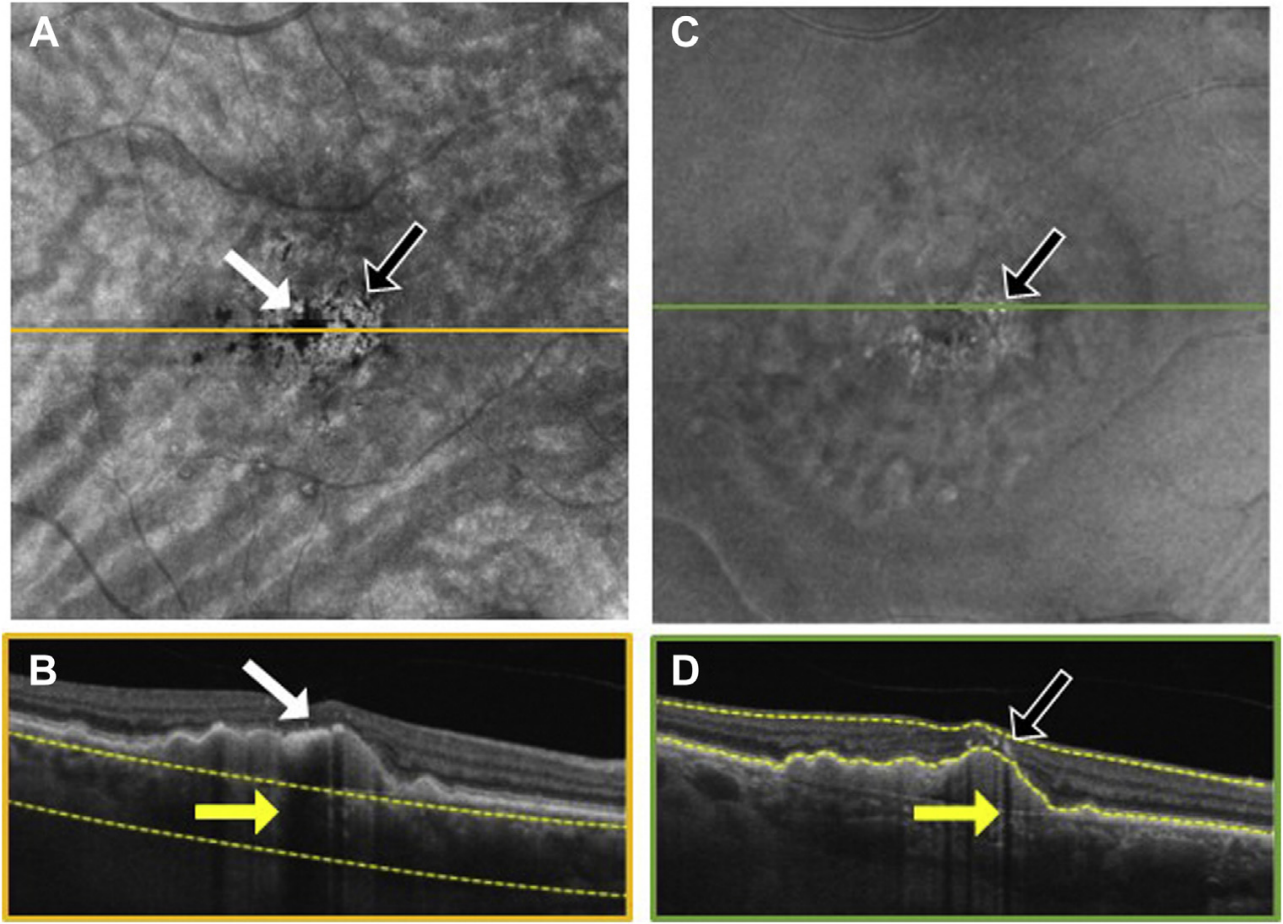
Swept-source OCT imaging of hyperpigmentation seen on color fundus images (CFI) from patient 1: (**A**) *en face* sub–retinal pigmented epithelium (RPE) slab, (**B**) B-scan corresponding to the orange line shown on the *en face* sub-RPE slab image (**A**) along with the yellow segmentation lines used to generate the sub-RPE slab image, (**C**) *en face* retinal slab, and (**D**) B-scan corresponding to the green line shown on the *en face* retinal image (**B**) along with the yellow segmentation lines used to generate the retinal slab. The hypotransmission defect (hypoTD) shown in (**A**) (white arrow) corresponds to regions of thickened RPE (**B**, white arrow) on top of a drusenoid pigmented epithelium detachment, and this hypoTD corresponds to the pigmentary changes previously seen on a CFI (Fig 1A). The hyperreflective foci (black arrow) seen in (**C**) also cause a hypoTD (**D**, yellow arrow) that can be appreciated as a dark spot in the sub-RPE *en face* image (**A**, black arrow). Once again, these hypoTDs were detected on the CFI as hyperpigmentation (Fig 1A, black arrow).

**Figure 4:**
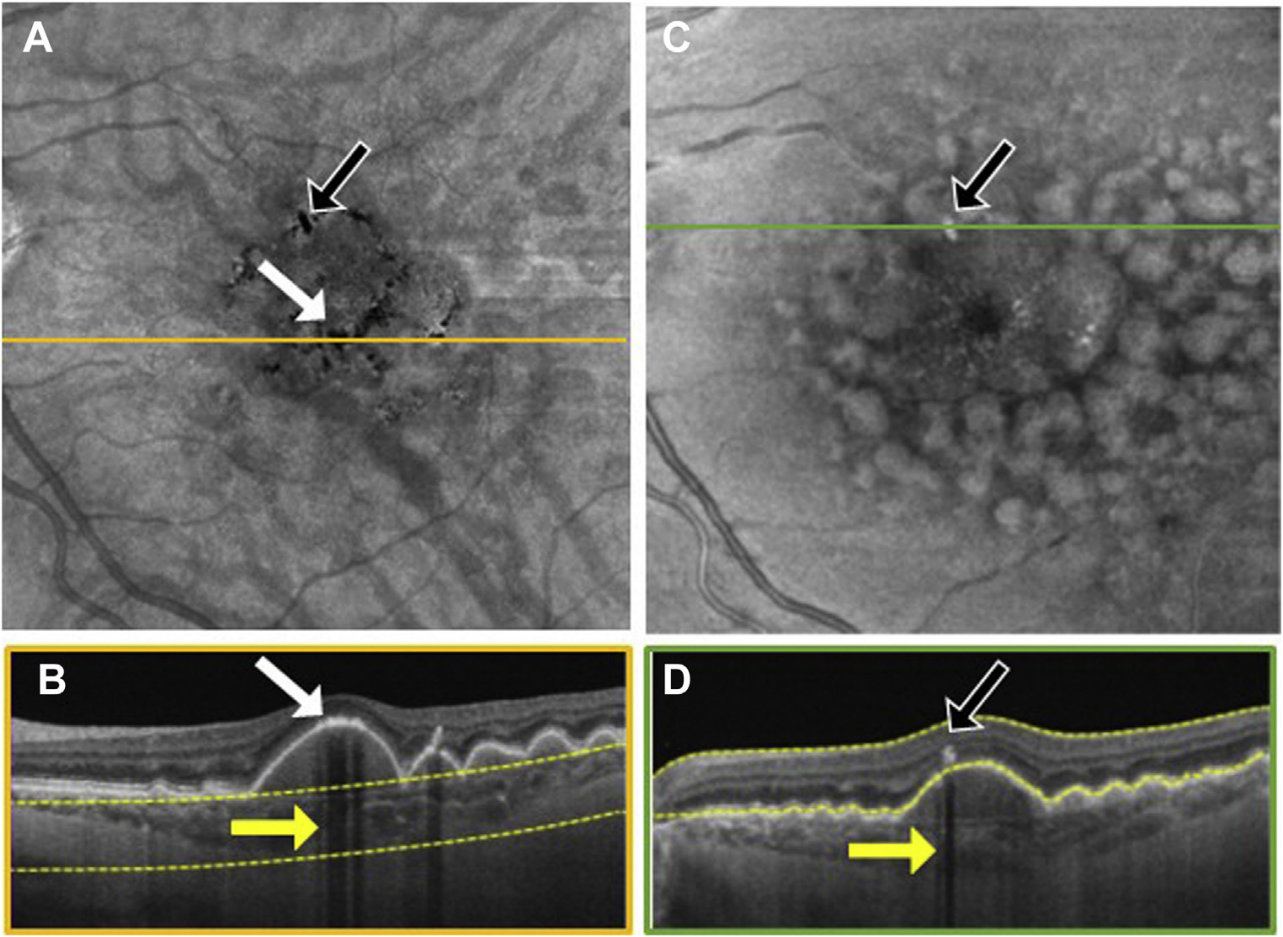
Swept-source OCT imaging of hyperpigmentation seen on color fundus images (CFI) from patient 2: (**A**) *en face* sub–retinal pigmented epithelium (RPE) slab, (**B**) B-scan corresponding to the orange line shown on the *en face* sub-RPE slab image (**A**) along with the yellow segmentation lines used to generate the sub-RPE slab image, (**C**) *en face* retinal slab, and (**D**) B-scan corresponding to the green line shown on the *en face* retinal image (**B**) along with the yellow segmentation lines used to generate the retinal slab. The hypotransmission defect (hypoTD) shown in (**A**) (white arrow) corresponds to regions of thickened RPE (**B**, white arrow) on top of a drusenoid pigmented epithelium detachment, and this hypoTD corresponds to the pigmentary changes previously seen in a CFI (Fig 1F). The hyperreflective foci (black arrow) seen in (**C**) also cause a hypoTD (**D**, yellow arrow) that can be appreciated as a dark spot in the sub-RPE *en face* image (**A**, black arrow). Once again, these hypoTDs were detected on the CFI as hyperpigmentation (Fig 1F, black arrow).

**Figure 5:**
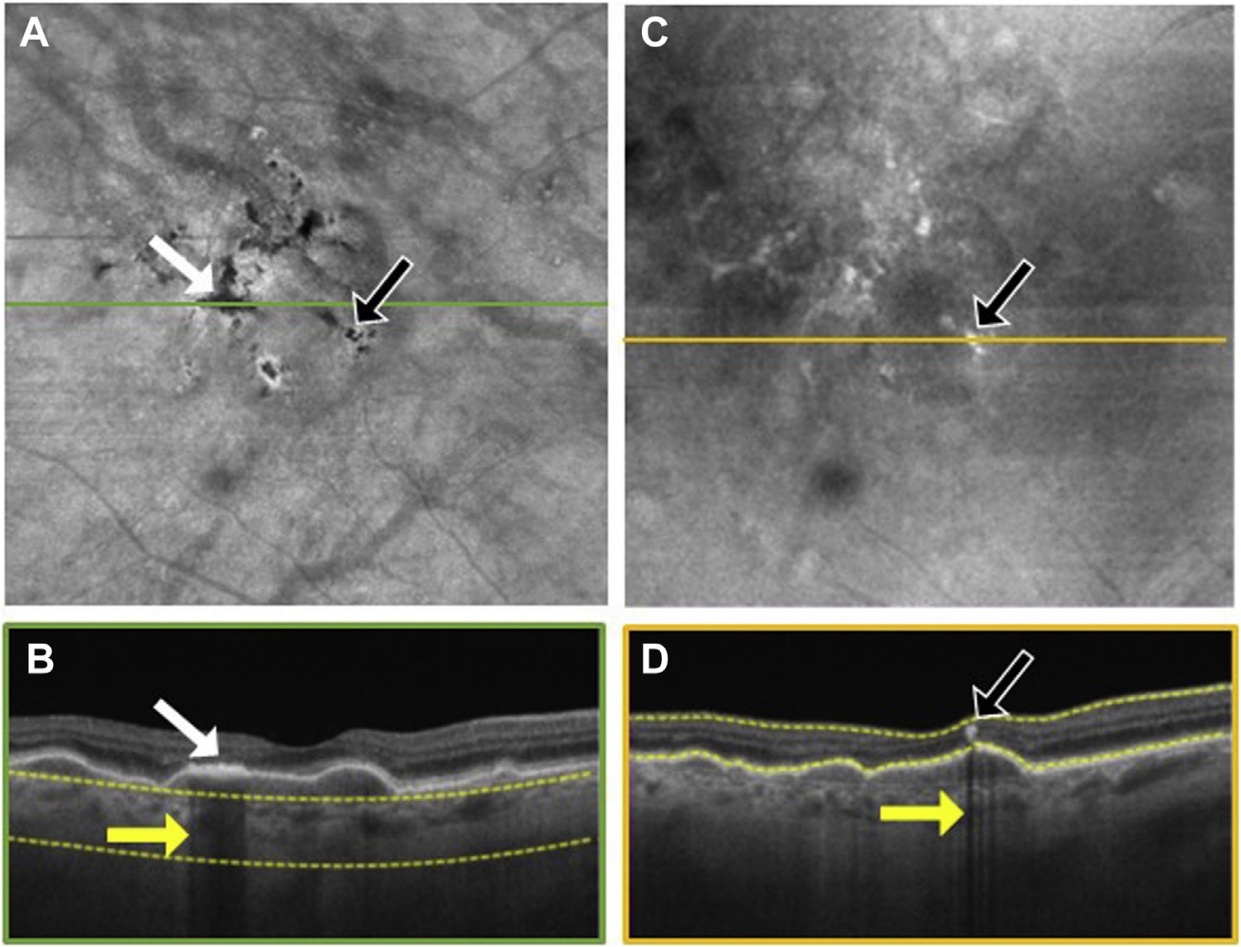
Swept-source OCT imaging of hyperpigmentation seen on color fundus images (CFI) from patient 3: (**A**) *en face* sub–retinal pigmented epithelium (RPE) slab, (**B**) B-scan corresponding to the orange line shown on the *en face* sub-RPE slab image (**A**) along with the yellow segmentation lines used to generate the sub-RPE slab image, (**C**) *en face* retinal slab, and (**D**) B-scan corresponding to the green line shown on the *en face* retinal image (**B**) along with the yellow segmentation lines used to generate the retinal slab. The hypotransmission defect (hypoTD) shown in (**A**) (white arrow) corresponds to regions of thickened RPE (**B**, white arrow) on top of a drusenoid pigmented epithelium detachment, and this hypoTD corresponds to the pigmentary changes previously seen in a CFI (Fig 1K). The hyperreflective foci (black arrow) seen in (**C**) also cause a hypoTD (**D**, yellow arrow) that can be appreciated as a dark spot in the sub-RPE *en face* image (**A**, black arrow). Once again, these hypoTDs were detected on the CFI as hyperpigmentation (Fig 1K, black arrow).

**Figure 6:**
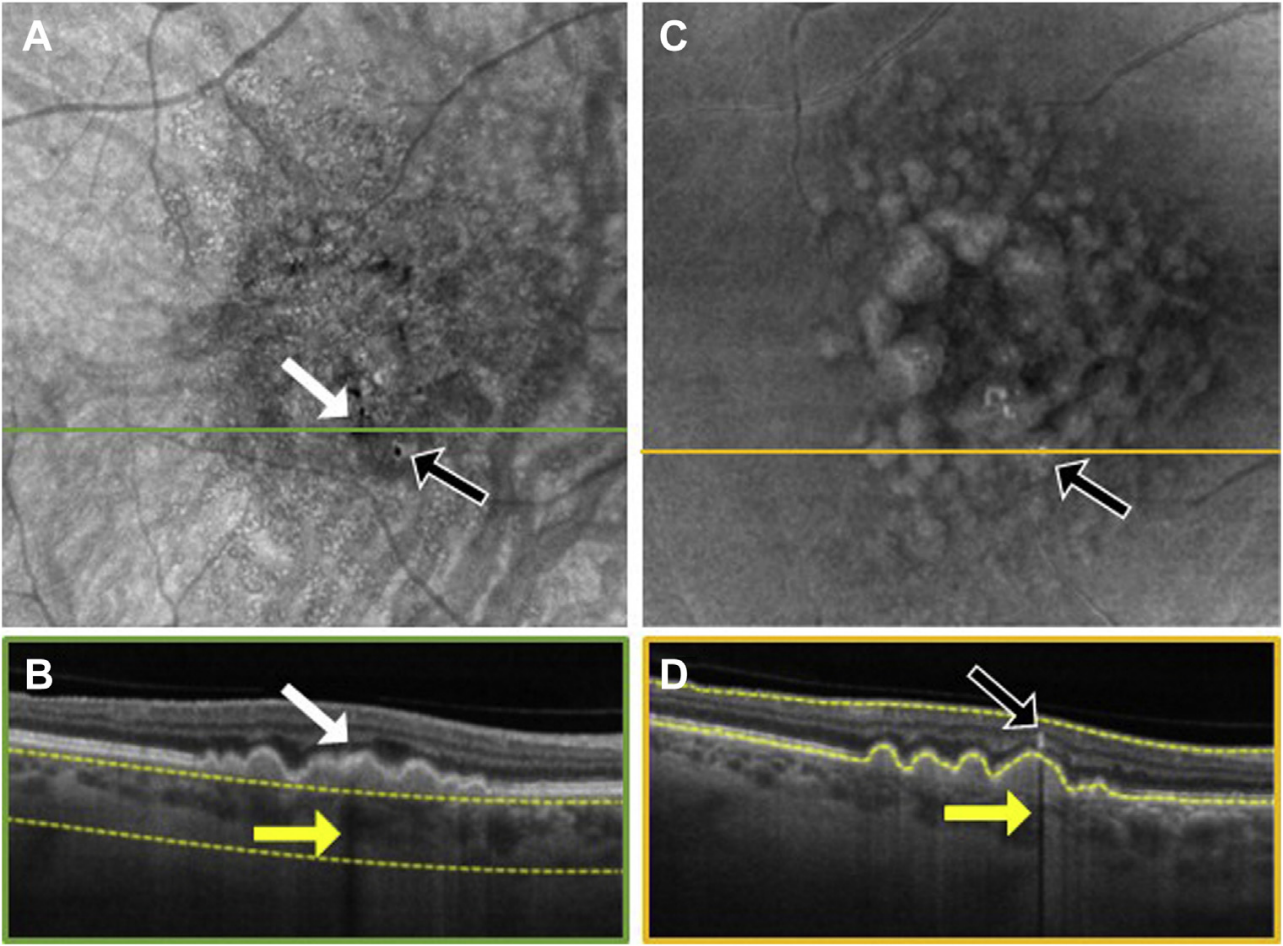
Swept-source OCT imaging of hyperpigmentation seen on color fundus images (CFI) from patient 4: (**A**) *en face* sub–retinal pigmented epithelium (RPE) slab, (**B**) B-scan corresponding to the orange line shown on the *en face* sub-RPE slab image (**A**) along with the yellow segmentation lines used to generate the sub-RPE slab image, (**C**) *en face* retinal slab, and (**D**) B-scan corresponding to the green line shown on the *en face* retinal image (**B**) along with the yellow segmentation lines used to generate the retinal slab. The hypotransmission defect (hypoTD) shown in (**A**) (white arrow) corresponds to regions of thickened RPE (**B**, white arrow) on top of a drusen, and this hypoTD corresponds to the pigmentary changes previously seen in a CFI (Fig 2A). The hyperreflective foci (black arrow) seen in (**C**) also cause a hypoTD (**D**, yellow arrow) that can be appreciated as a dark spot in the sub-RPE *en face* image (**A**, black arrow). Once again, these hypoTDs were detected on the CFI as hyperpigmentation (Fig 2A, black arrow).

**Figure 7:**
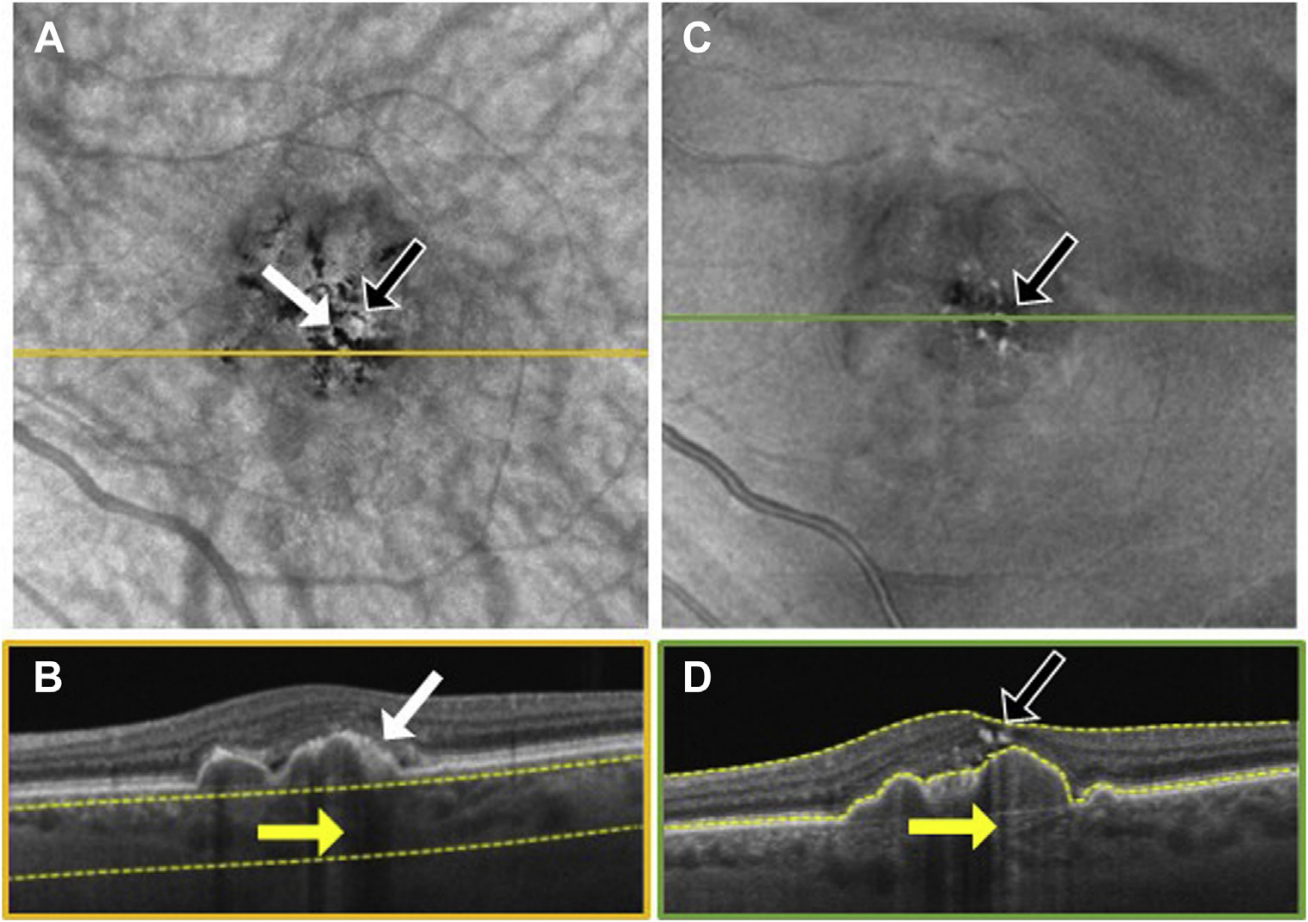
Swept-source OCT imaging of hyperpigmentation seen on color fundus images (CFI) from patient 5: (**A**) *en face* sub–retinal pigmented epithelium (RPE) slab, (**B**) B-scan corresponding to the orange line shown on the *en face* sub-RPE slab image (**A**) along with the yellow segmentation lines used to generate the sub-RPE slab image, (**C**) *en face* retinal slab, and (**D**) B-scan corresponding to the green line shown on the *en face* retinal image (**B**) along with the yellow segmentation lines used to generate the retinal slab. The hypotransmission defect (hypoTD) shown in (**A**) (white arrow) corresponds to regions of thickened RPE (**B**, white arrow) on top of a drusenoid pigmented epithelium detachment, and this hypoTD corresponds to the pigmentary changes previously seen in a CFI (Fig 2F). The hyperreflective foci (black arrow) seen in (**C**) also cause a hypoTD (**D**, yellow arrow) that can be appreciated as a dark spot in the sub-RPE *en face* image (**A**, black arrow). Once again, these hypotransmission defects were detected on the CFI as hyperpigmentation (Fig 2F, black arrow).

**Figure 8:**
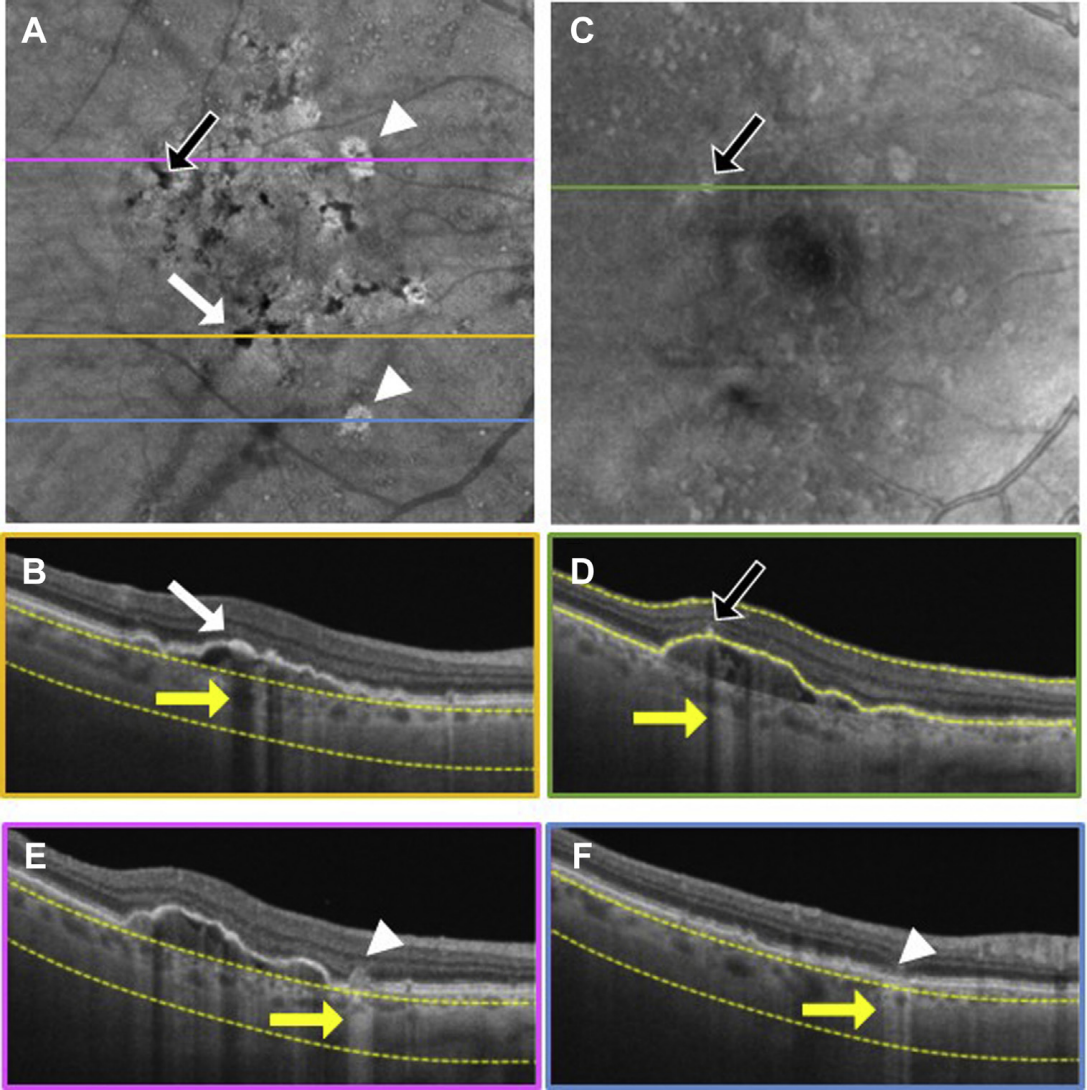
Swept-source OCT imaging of hyperpigmentation seen on color fundus images (CFI) from patient 6: (**A**) *en face* sub–retinal pigmented epithelium (RPE) slab, (**B**) B-scan corresponding to the orange line shown on the *en face* sub-RPE slab image (**A**) along with the yellow segmentation lines used to generate the sub-RPE slab image, (**C**) *en face* retinal slab, (**D**) B-scan corresponding to the green line shown on the *en face* retinal image (**B**) along with the yellow segmentation lines used to generate the retinal slab, (**E**) B-scan corresponding to the pink line shown on the *en face* retinal image (**B**) along with the yellow segmentation lines used to generate the retinal slab, and (**F**) B-scan corresponding to the blue line shown on the *en face* retinal image (**B**) along with the yellow segmentation lines used to generate the retinal slab. The hypotransmission defect (hypoTD) shown in (**A**) (white arrow) corresponds to regions of thickened RPE (**B**, white arrow) on top of a drusenoid pigmented epithelium detachment, and this hypoTD corresponds to the pigmentary changes previously seen in a CFI (Fig 2K). The hyperreflective foci (black arrow) seen in (**B**) also cause a hypoTD (**D**, yellow arrow) that can be appreciated as a dark spot in the sub-RPE *en face* image (**A**, black arrow). Once again, these hypoTDs were detected on the CFI as hyperpigmentation (Fig 2K, black arrow). The hypertransmission defects (hyperTDs) can also be appreciated in the sub-RPE slab as bright spots that are clearly distinguishable from the pigmentary changes (**A**, white arrowheads). These hyperTDs correspond in the B-scans to regions of absent or damaged RPE associated with a collapsing druse (**E**, white arrowhead pointing to the druse and yellow arrow showing the hypertransmission of light into the choroid) and to regions of RPE and outer retinal atrophy (**F**, white arrowhead pointing to RPE and outer retinal atrophy, yellow arrow showing hypertransmission of light into the choroid).

To explore the location of hyperpigmentation seen as dark spots on the SS OCT *en face* sub-RPE images and bright spots on the *en face* retinal slabs, we reviewed the corresponding B-scans (Figure 3, Figure 4, Figure 5, Figure 6, Figure 7, Figure 8). In Figure 3, Figure 4, Figure 5, Figure 6, Figure 7, Figure 8, the white arrows in (A) correspond to regions in which the RPE appeared thickened, and this structural change appeared to attenuate the penetration of light into the choroid (Figs 3B–8B, white arrows) causing a hypoTD (Figs 3B–8B, yellow arrows). We also showed that the bright spots seen in the SS OCT *en face* retinal slab (Figs 3C–8C, black arrows) corresponded to hyperreflective foci in the retina (panel D, black arrows). In addition, these hyperreflective foci could also cause attenuation of light into the choroid, which also resulted in hypoTDs (Figs 3D–8D, yellow arrows) that were detected in the sub-RPE *en face* images (Figs 3A–8A, black arrows). In Figure 8E, F, the brighter areas seen on the sub-RPE slab are shown to correspond to B-scan hyperTDs, once again demonstrating the benefits of the sub-RPE slab in identifying areas of evolving atrophy.

## Discussion

In this study, we demonstrated that *en face* OCT imaging provides a reliable and easy-to-use tool to detect hyperpigmentation seen in CFIs in eyes with iAMD. Using 2 *en face* slabs, the retinal slab and the sub-RPE slab, we were able to easily identify all macular pigmentary changes. The retinal slab identified hyperreflective foci within the retina that are thought to represent RPE migration.^14^ The sub-RPE slab identified regions of a thickened RPE that blocked the penetration of light into the choroid, causing hypoTDs. Most importantly, we showed that hyperreflective foci in the retina represent only a fraction of the hyperpigmentation seen on CFIs, and macular hyperpigmentation should not be considered synonymous with these intraretinal hyperreflective foci. In addition, not all hypoTDs seen on the sub-RPE slabs arose from the RPE layer, because large intraretinal hyperreflective foci can also cast a shadow onto the sub-RPE slab when these intraretinal hyperreflective foci are adjacent to the RPE. These findings are relevant to eyes with iAMD and may be useful for identifying those eyes at risk for disease progression; however, as eyes progress to late AMD, additional changes occur, such as macular neovascularization, exudates, and hemorrhages, that can cause hypoTDs on *en face* images.

In the current study, we performed an analysis of different imaging methods to detect macular hyperpigmentation, with CFIs serving as the gold standard. Blue-light FAF imaging failed to detect many of the pigmentary abnormalities, whereas NIR imaging detected most of the pigmentary changes seen on CFIs. The usefulness of NIR suggests that it is the melanin in these pigmentary abnormalities that is responsible for their detection using both infrared reflectance^21^^,^^22^ and OCT imaging. However, NIR imaging has a disadvantage when evaluating hyperpigmentation in the context of drusen-related atrophy. In this situation, both pigmentary clumps and early atrophic lesions appear as bright lesions (Fig 2M). Areas of atrophy appear as hyperreflective patches on NIR imaging because of the absence of blockage from the RPE and increased reflection of light from the sclera.^23^ Thus, the distinction between hyperpigmentation and early atrophy using NIR imaging is dependent on a second examination such as OCT imaging. *En face* structural OCT imaging seemed to be the most reliable method for detecting the hyperpigmentation seen in CFIs. Using 2 *en face* OCT slabs from the same scan pattern, we were able to detect not only the intraretinal hyperreflective foci using the retinal slab, but also the hypoTDs seen on the sub-RPE slab that corresponded to blockage of the light into the choroid in regions where the RPE was thickened. The findings on both slabs from the same scan correlated with regions of hyperpigmentation seen in CFIs. OCT *en face* imaging had the additional advantage of allowing a clear distinction between hyperpigmentation seen as hypoTDs (dark spots) and early atrophic lesions seen as hyperTDs (bright spots; Fig 2N). Moreover, the advantages of using OCT *en face* imaging compared with individual B-scans alone include the ability to review an *en face* image rapidly for the detection of pathologic features and the ability to view the disease in 2 lateral dimensions with the ability to promptly correlate the *en face* findings in the depth dimension by using the structural B-scans. In addition, the use of *en face* OCT imaging and its unambiguous interpretation of bright and dark spots allows for the development of automated algorithms to quantify the burden of these areas of hyperpigmentation, to assess disease severity, and to predict disease progression. If the burden of these pigmentary abnormalities can be easily quantified, then it would alleviate the laborious task of manually reviewing of all B-scans and assessing the number and size of these hyperreflective foci in the B-scans and along the RPE.

The ability to identify intraretinal hyperreflective foci and abnormal thickening of the RPE as causes of macular hyperpigmentation may increase the ability to use OCT alone to assess disease severity and the risk of progression.^20^^,^^24^ The use of *en face* images in conjunction with B-scan location of hyperTDs and hypoTDs provides a 3-dimensional view of macular anatomic features that should prove useful when developing risk scores for disease severity and progression and should improve recent OCT-based methods for predicting AMD progression.^24^^,^^25^

We are aware that our study is limited by its small sample size. However, we believe that this preliminary case series is useful to reinforce the concept that hyperpigmentation in the macula arises not only from hyperreflective foci in the retina, but also from areas of increased pigmentation along the RPE layer,^15^ and our observations were obtained using commercially available OCT imaging strategies. These observations show the importance of developing new automated algorithms that can quantify this increased pigmentation in the retina and along the RPE. Such a pigment-burden algorithm could be used, together with existing algorithms, to assess drusen area and volume^26^ to assess the risk of disease progression in eyes with iAMD. This should prove useful when identifying eyes at risk for drusen-related atrophy that could be enrolled into clinical trials to test novel therapies to prevent disease progression from iAMD to late AMD.

In summary, we used multimodal imaging to identify and characterize the hyperpigmentation seen in CFIs in eyes with iAMD. We showed that *en face* OCT imaging is a reliable method for clinically identifying and localizing the hyperpigmentation seen in CFIs. Although hyperreflective foci have been used as a surrogate for the hyperpigmentation, we showed that they did not correspond to all the macular hyperpigmentation seen on CFIs. The HypoTDs that are associated with regions of thickened RPE that block the penetration of light into the choroid are an important component of the macular hyperpigmentation and may be even more important than hyperreflective foci in predicting disease progression in clinical practice. Natural history studies are underway to assess the importance of both hyperreflective foci in the retina and hypoTDs associated with thickened areas of the RPE in predicting the progression from iAMD to late AMD.
